# Lead exposure at firing ranges—a review

**DOI:** 10.1186/s12940-017-0246-0

**Published:** 2017-04-04

**Authors:** Mark A. S. Laidlaw, Gabriel Filippelli, Howard Mielke, Brian Gulson, Andrew S. Ball

**Affiliations:** 1 Centre for Environmental Sustainability and Remediation (EnSuRe), School of Science, RMIT University, PO Box 71, Bundoora, VIC 3083 Australia; 2 Department of Earth Sciences and Center for Urban Health, Indiana University-Purdue University Indianapolis (IUPUI), Indianapolis, IN USA; 3 Tulane University School of Medicine, New Orleans, LA USA; 4 Department of Environmental Sciences, Macquarie University, Sydney, Australia

**Keywords:** Blood, Lead, Poisoning, Shooting, Range, Firearms, Health, Effects, Expert shooter, Guns

## Abstract

**Background:**

Lead (Pb) is a toxic substance with well-known, multiple, long-term, adverse health outcomes. Shooting guns at firing ranges is an occupational necessity for security personnel, police officers, members of the military, and increasingly a recreational activity by the public. In the United States alone, an estimated 16,000–18,000 firing ranges exist. Discharge of Pb dust and gases is a consequence of shooting guns.

**Methods:**

The objectives of this study are to review the literature on blood lead levels (BLLs) and potential adverse health effects associated with the shooting population. The search terms “blood lead”, “lead poisoning”, “lead exposure”, “marksmen”, “firearms”, “shooting”, “guns”, “rifles” and “firing ranges” were used in the search engines Google Scholar, PubMed and Science Direct to identify studies that described BLLs in association with firearm use and health effects associated with shooting activities.

**Results:**

Thirty-six articles were reviewed that included BLLs from shooters at firing ranges. In 31 studies BLLs > 10 μg/dL were reported in some shooters, 18 studies reported BLLs > 20 μg/dL, 17 studies > 30 μg/d, and 15 studies BLLs > 40 μg/dL. The literature indicates that BLLs in shooters are associated with Pb aerosol discharge from guns and air Pb at firing ranges, number of bullets discharged, and the caliber of weapon fired.

**Conclusions:**

Shooting at firing ranges results in the discharge of Pb dust, elevated BLLs, and exposures that are associated with a variety of adverse health outcomes. Women and children are among recreational shooters at special risk and they do not receive the same health protections as occupational users of firing ranges. Nearly all BLL measurements compiled in the reviewed studies exceed the current reference level of 5 μg/dL recommended by the U.S. Centers for Disease Control and Prevention/National Institute of Occupational Safety and Health (CDC/NIOSH). Thus firing ranges, regardless of type and user classification, currently constitute a significant and unmanaged public health problem. Prevention includes clothing changed after shooting, behavioural modifications such as banning of smoking and eating at firing ranges, improved ventilation systems and oversight of indoor ranges, and development of airflow systems at outdoor ranges. Eliminating lead dust risk at firing ranges requires primary prevention and using lead-free primers and lead-free bullets.

**Electronic supplementary material:**

The online version of this article (doi:10.1186/s12940-017-0246-0) contains supplementary material, which is available to authorized users.

## Background

Most attention in the area of human health and guns has been rightly placed on shooting injuries and deaths [1]. However, decades of evidence indicate that substantial health risks are incurred by the shooters themselves in the form of lead exposure and subsequent poisoning. Indeed, as pointed out as early as 1994 by Ozonoff, based on high blood lead levels (BLLs) of shooters, “…firing ranges comprise one of the largest unregulated sources of occupational or para-occupational lead exposure for adults. The perils of firearms exist at both ends of the barrel.” [2]. The past two decades have brought substantial improvements in firing range environmental oversight as well as analytical capabilities to detect lead in humans, but literature evidence indicates that we fall far short of human health safety criteria in firing ranges of all types, and among occupational and recreational shooters. This review fills a gap in the literature by compiling data from a broad range of recent studies of firing range users, employees, and their families, including indoor and outdoor ranges, in an attempt to document and clarify risks by firing range use, setting, and shooting behaviour. The emphasis of this review is on BLLs of shooters as a marker for adverse health effects among members of the shooting population.

### Shooting statistics

In the United States alone an estimated 16,000–18,000 indoor firing ranges exist which employ tens of thousands of employees [3]. An estimated 1 million law enforcement officers train at indoor firing ranges [3]. In 2011 there were approximately 270 million civilian-owned firearms owners in the US and in 2007 there were approximately 650 million civilian-owned weapons globally [4] and 200 million firearms owned by nation states worldwide [5]. In the United States approximately 20 million citizens practice target shooting as a leisure activity [6]. The National Sport Shooting Foundation (NSSF) [7] stated that in 2011 in the United States there were 13,049,050 handgun shooters, 13,170,417 rifle shooters, 9,713,033 shotgun shooters, and 3,730,567 muzzleloader shooters who participated in 156,790,412 handgun shooting days, 146,652,398 rifle shooting days, 113,866,661 shotgun shooting days and 29,042,237 muzzleloader shooting days. The global statistics regarding the number of firing ranges and shooting prevalence are not available, but it is likely there are a very large number of shooters at firing ranges. The United States Geological Survey (USGS) [8] calculated that in 2012 about 60,100 metric tonnes of lead were used in ammunition and bullets in the United States. Given that the dominant metal in bullets is lead, there are a large number of people globally who are exposed to lead from shooting at firing ranges.

### Source of exposure from shooting lead bullets

There are several sources of potential lead exposure from shooting guns and firing ranges. Most bullet projectiles are made from lead, but a large amount of lead is also present in the primer, composed of approximately 35% lead styphnate and lead peroxide (and also contains barium and antimony compounds), that ignites in a firearm barrel to provide the propulsion for the projectile [9–13]. A portion of the lead bullet disintegrates into fine fragments while passing through the gun due to misalignments of the gun barrel [9]. The lead particles, along with dust and fumes originating from the lead primer and the bullet fragments are ejected at high pressures (18,000–20,000 psi; 124–128 mpa) from the gun barrel, a large proportion of which occurs at right angles to the direction of fire in close proximity to the shooter [9]. The shooter can inhale fine Pb particulates (mainly from the primer) which constitutes the proximal exposure pathway. Fine and coarse particulates from both the primer and bullet fragments also attach to the shooters hands, clothing, and other surfaces, and can be inadvertently ingested, providing another lead exposure pathway [14, 15]. When changing targets at outdoor firing ranges shooters can be exposed to lead that has accumulated in soil dust. Additionally, the shooters can then bring these particulates back to their home and expose their families as with other lead occupational hazards.

Firing range personnel are employed by the shooting galleries, and thus also receive proximal lead exposure. They are also charged with cleaning the ranges and removing lead particulates on floors, targets, and the ventilation systems (for indoor ranges). Furthermore, they work at firing ranges for extended hours during the working week, compounding potential lead exposure.

Finally, although not the focus of this review, there are environmental impacts arising from firing ranges. Emissions in firing ranges result in the accumulation of elevated lead concentrations in surface soils [16–18]. This is concerning because lead particles do not naturally biodegrade in soil as do some contaminants such as hydrocarbons. The half-life of lead in surface soil has been estimated as approximately 700 years [19]. Therefore, if not remediated following closure, lead contaminated surface soils at firing ranges could result in lead exposures for hundreds of years. Dust from lead contaminated soil can be resuspended into the atmosphere and transported from a firing range whether outdoor or indoor [20, 21]. Lead in soils and dusts at firing ranges are highly bioavailable [22]. Lead in soil could weather/oxidize and migrate down-gradient to underlying groundwater beyond the firing range boundaries [23]. The low solubility of lead in natural water (i.e., not mining related), however, limits off-site aquatic transport. The factors most likely to affect the amount of lead carried by the groundwater in solution are pH, depth to groundwater, soil chemistry, soil type and annual precipitation [24]. Soil-derived sediment discharged during rain events from lead contaminated firing range soils has the potential to migrate to surrounding properties and into waterways through runoff or storm drains. Wildlife [25–27], biota [28] and humans can be exposed to lead contaminated soils, sediments and airborne particulates near firing ranges. Bellinger et al. (2013) [29] provided a consensus statement about the health risks arising from lead-based ammunition in the environment.

### Health outcomes associated with blood lead levels (BLLs)

In 2012, The United States National Toxicology Program (NTP) published evidence regarding health effects associated with BLL exposure in adults and children [30]. For adult men and women there is “sufficient evidence” that BLLs <10 μg/dL are associated with essential tremor, hypertension, cardiovascular-related mortality and electrocardiography abnormalities, and decreased kidney glomerular filtration rate. For women there is “sufficient evidence” that BLLs <5 μg/dL are associated with reduced foetal growth. For adult men and women there is “limited evidence” that BLLs <10 μg/dL were associated with psychiatric effects, decreased hearing and cognitive function, incidence of amyotrophic lateral sclerosis, and increased spontaneous abortion in women. For adults there is “limited evidence” that BLLs <5 μg/dL were associated with incidence of essential tremor. For children with BLLs <5 μg/dL there is “sufficient evidence” of decreased academic achievement, intelligence quotient (IQ), specific cognitive measures, increased attention related behaviours, delayed puberty and reduced postnatal growth. For children with blood lead levels < 10 μg/dL there is “sufficient evidence” of decreased hearing. For children with BLLs < 5 μg/dL there is “limited evidence” of an association of decreased kidney glomerular filtration rate, and delayed puberty. For prenatal exposure with BLLs < 5 μg/dL there is “limited evidence” of decreases in measures of cognitive function. For prenatal lead exposure < 10 μg/dL there is “limited evidence” of decreased IQ, increased incidence of attention-related and problem behaviors and decreased hearing. For adult men and women there is “limited evidence” that BLLs between 15 and 20 μg/dL are associated with adverse sperm parameters and increased time to pregnancy in women. There is “limited evidence” that BLLs ≥10 μg/dL are associated with decreased fertility. There is “limited evidence” that spontaneous abortion occurs in female partners of men with BLLs ≥ 31 μg/dL. However, modern exposures are orders of magnitude larger than early hominids [31] with pre-industrial blood lead levels in humans estimated at 0.016 μg/dL [32]. Bellinger (2011) [33] noted that adverse health effects are continually being associated with decreasing exposures.

## Methodology

The search engines Google Scholar, Pubmed and Science Direct were accessed for studies that provided information about BLLs associated with firearms using the search terms “blood lead”, “lead poisoning”, “lead exposure”, “marksmen”, “firearms”, “shooting”, “guns”, “rifle” and “firing ranges”. The literature regarding the health effects specifically associated with shooting lead bullets at firing ranges was also reviewed. Studies that reported BLLs associated with shooters at firing ranges were compiled into Table 1.

**Table 1:** Blood Lead Levels Observed Following Shooting Firearms at Shooting ranges

			Blood Lead (μg/dL)					
Activity	Firing		Mean (Median*)	SD	Range			
Type	Range	Category				Location	n	Reference
Occupational								
	Indoor							
		Arsenal Firing Range Workers	37.2	11.7	22–59.6	Taiwan	10	Chau et al. (1995) [80]
		Arsenal Other Workers	13.3	5.6			98	
		Firing Range and Gun Store Employees			19.9–40.7	California, USA	6	CDC (2014) [81]
		Firearms instructor & range cleaning and maintenance	88			New York, USA	1	Fisher-Fischbein et al. (1987) [82]
		Firearm Instructors Before Indoor Training: 4.3%			<20	New York, USA	1	Fischbein et al. (1979) [36]
		Firearm Instructors Before Indoor Training: 82.6%			20–39		19	
		Firearm Instructors Before Indoor Training: 8.7%			40–59		2	
		Firearm Instructors Before Indoor Training: 4.3%			>60		1	
		Firearm Instructors After Indoor Training: 4.3%			<20		1	
		Firearm Instructors After Indoor Training: 52.2%			20–39		12	
		Firearm Instructors After Indoor Training: 34.8%			40–59		8	
		Firearm Instructors After Indoor Training: 8.7%			>60		2	
		Firearms Instructors and Officers: 42%			<40	New York, USA	19	Fischbein (1992) [83]
		Firearms Instructors and Officers: 34%			40–60		15	
		Firearms Instructors and Officers: 24%			>60		11	
		Firearm Instructors	125	15		USA	3	Landrigan et al. (1975) [84]
		Male Police Officers	5		1–18.2	Sweden	75	Löfstedt et al. (1999) [85]
		Female Police Officers	3.7				3	
		Full Time Employee	77			Colorado, USA	1	Novotny et al. (1987) [86]
		Full Time Employee	59				1	
		Full Time Employee	49				1	
		Part Time Employee	41				1	
		Shooting range supervisor	5.8	1.6	2.1–17.3	Republic of Korea	35	Park et al. (2016) [87]
		Professional shooter	11.6	1.9	3.0–34.3		24	
		Shooting range manager	9.5	2.2	2.0–64.0		61	
		All job types	8.6	2.1	2.0–64.0		120	
		Firing Range Instructors	5.5	0.6	4.2–6.7	Sao Paulo, Brazil	20	Rocha et al. (2014) [35]
		Cadets - Before Training	3.3	0.15	3.0–3.6		21	
		Cadets - After Training	18.2	1.5	16–21		21	
		Instructor 1 Before Training	3.6				1	
		Instructor 1 After Training	22.1				1	
		Instructor 2 Before Training	7.7				1	
		Instructor 2 After Training	18.3				1	
		Instructors	19	7		Amman, Jordan	31	Abudhaise et al. (1996) [88]
		Trainees	22.9	4.6			54	
		Controls	2.1	1.4			unknown	
		Police Small Arms Instructors	43.5	6.2	33.1–49.7	England	7	Smith (1976) [89]
		US Coastguard Firearm Instructors - Pre BLL	2.4	1.6	0.9–5.6	Washington, USA	9	Torres (2014) [90]
		US Coastguard Firearm Instructors - Post BLL	2.3	1.2	0.9–4.6		9	
		US Coastguard Firearm Instructors - Pre-Post BLL	2.4	1.4	0.9–5.1		9	
		Police Trainees: Before Training - (1/29/87)	6.0 (6.4*)			Colorado, USA	17	Valway et al. (1989) [45]
		Police Trainees: Training - (3/3/87)	51.1 (51.6*)				17	
		Police Trainees: Training - (3/31/87)	44.3 (43.1*)				17	
		Police Trainees: Training - (5/3/87)	39.5 (40.0*)				17	
		Israeli Defence Soldiers Baseline	10.3	2.3		Israel	29	Vivante et al. (2008) [37]
		Israeli Defence Soldiers Postexposure	18.8	3.6			29	
		Indoor Range Instructors	8.5	7.6		Italy	unknown	Di Lorenzo et al. (2006) [91]
	Indoor/outdoor							
		FBI Instructors 1989	14.6		9–21	Virginia, USA	7	CDC (1996) [65]
		FBI Instructors 1990	13.7		6–27		7	
		FBI Instructors 1991	7.6		<4–12		14	
		FBI Range Techs 1989	16.2		10–24		5	
		FBI Range Techs 1990	10.4		6–14		5	
		FBI Range Techs 1991	13.6		8–28		5	
		US Special Operations Forces - Year 2000	13.90			USA	255	Mancuso et al. (2008) [92]
		US Special Operations Forces - Year 2005	6.80					
		Adult Target Shooters - Occupational - 21.6%			<25	New York, USA	26	Gelberg and DePersis (2009) [93]
		Adult Target Shooters - Occupational - 50.8%			25–39		61	
		Adult Target Shooters - Occupational - 25%			40–59		30	
		Adult Target Shooters - Occupational - 2.5%			60+		3	
	Outdoor							
		Range Instructors	6.7	5.3		Italy	unknown	Di Lorenzo et al. (2006) [91]
		Firing Range Instructors Before Training	41	10	28–66	California, USA	7	Goldberg et al. (1991) [38]
		Firing Range Instructors After Training	45	14	28–70		7	
		Firing Range Instructors 6 Months After Training	31	5	28–38		7	
		Soldiers Before Basic Training	<0.29			Israel	22	Greenberg et al. (2016) [94]
		Soldiers After Basic Training	1.17	1.73			22	
		Soldiers After Advanced Training	3.92	1.99			22	
		Body guards	8.10	2.70	3.0–14.4	Mexico	26	Madrid et al. (2016) [41]
		Instructors	7.60	1.40	6.6–8.6		2	
		Shooting Range Caretakers	40.65	15.63	29.6–51.7		2	
		Administrative	4.20	1.20	2.7–6.5		9	
		Security	4.90	1.20	3.0–7.3		9	
		Services	6.50	3.60	3.1–14.2		10	
		Elite Group	6.40	2.60	4.2–11.4		6	
		All Categories	7.60	6.80	2.7–51.7		64	
		Howitzer Operators - Baseline	5.5		4.33–6.73	Oklahoma, USA	20	Smart et al. (1994) [40]
		Howitzer Operators - After 3rd Exercise	20.1		17.94–22.44		20	
		Howitzer Operators - 8 Weeks After Exposure	11.9		9.92–14.02		12	
		Shooting Instructor #1 Non-Jacketed bullets	24.02			Virginia, USA	1	Tripathi et al. (1991) [46]
		Shooting Instructor #1 Copper Jacketed Bullets	21.95				1	
		Shooting Instructor #2 Non-Jacketed bullets	14.08				1	
		Shooting Instructor #2 Copper Jacketed Bullets	13.04				1	
		Outdoor Firing Range Police Cadets: Baseline - Day 0	6	1.1	5–8	Virginia, USA	7	Tripathi et al. (1989) [9]
		Outdoor Firing Range Police Cadets: Day 1	6	1.0	5–8		7	
		Outdoor Firing Range Police Cadets: Day 2	11	2.1	7–14		7	
		Outdoor Firing Range Police Cadets: Day 5	15	5.4	9–26		7	
		Outdoor Firing Range Police Cadets: Day 69	9	2.8	6–14		7	
Non-Occupational								
	Indoor							
		High School Shooting Coach	44			Alaska, USA	1	CDC (2005) [56]
		Firing Range A (School Range) - Students Aged 15–17 years	24.3		21.0–31.0		7	
		Firing Range B (Commercial) - Students Aged 13–16 years	2.1				8	
		Firing Range C (Volunteer Run) - Students Aged 15–19 years	18.5		5–37		24	
		Firing Range D (School Range) - Students Aged 14–17 years	8.9		3.0–14.0		7	
		Firing Range E (Volunteer Run) - Students Aged 7–17 years	7.6		2–13		20	
		Shooter	9.2*		2.7–52.1	Germany	129	Demmeler et al. (2009) [44]
		Shooter: Q1 200 rounds/month	8.7*		2.8–31.4		27	
		Shooter: Q2 200–399 rounds/month	9*		2.7–31.5		28	
		Shooter: Q3 400–680 rounds/month	11.8*		2.9–37.5		29	
		Shooter: Q4 > 680 rounds/month	13.8*		3.7–52.1		23	
		Airguns	3.3*		1.8–12.7		20	
		Airguns and .22 caliber	8.7*		1.4–17.2		15	
		.22 lr bore & large caliber	10.7*		2.7–37.5		51	
		Large caliber handgun	10*		2.8–32.6		32	
		IPSC (International Practical Shooting Federation)	19.2*		3.2–52.1		11	
		Adult Target Shooters - Both Occupational & Non-Occupational - 1.5%			<25	New York, USA	2	Gelberg and DePersis (2009) [93]
		Adult Target Shooters - Both Occupational & Non-Occupational - 71.3%			25–39		92	
		Adult Target Shooters - Both Occupational & Non-Occupational - 20.9%			40–59		27	
		Adult Target Shooters - Both Occupational & Non-Occupational - 6.2%			60+		8	
		Small Bore Rifle Shooter - End of Indoor Season	54.65			New Zealand	52	George et al. (1993) [39]
		Small Bore Rifle Shooter - Preseason Following Year	33.13				37	
		Shooting Range #2	10.5	7		South Africa	30	Mathee et al. (2017) [15]
		Shooting Range #3	16.1	9.8			17	
		Shooting Range #4	19.2	16.3			14	
		Child (Age 1 year) - “index case”	46			USA	1	Moore (1995) [95]
		Mother - (Age 25 years)	11				1	
		Father - (Age 25 years)	15				1	
		Sibling - (Age 4)	14				1	
		Sibling - (Age 2)	19				1	
		Cousin - (Age 6)	28				1	
		Cousin - (Age 8)	31				1	
		Aunt - (Age 37)	30				1	
		Child (Age 1 year.) “index case”: 3 Months After Intervention	19				1	
		Mother - (Age 25 years): 3 Months After Intervention	8				1	
		Father - (Age 25 years): 3 Months After Intervention	Not Measured				1	
		Sibling - (Age 4): 3 Months After Intervention	11				1	
		Sibling - (Age 2): 3 Months After Intervention	12				1	
		Cousin- (Age 6): 3 Months After Intervention	13				1	
		Cousin - (Age 8): 3 Months After Intervention	13				1	
		Aunt - (Age 37): 3 Months After Intervention	13				1	
		Indoor Shooting Range Employee Spouse	6			Colorado, USA	1	Novotny et al. (1987) [86]
		Indoor Shooting Range Employee Spouse	11				1	
		Indoor Shooting Range Employee Spouse	6				1	
		Recreational Shooters	29*		24–45	Germany	7	Ochsmann et al. (2009) [6]
		Competitive Marksmen - Adolescents Aged 14–16 years	21.3		18–28	Massachusetts, USA	4	Shannon (1999) [57]
		Shooters before indoor shooting season	10.6*		3.2–17.6	Sweden	22	Svensson et al. (1992) [96]
		Shooters after indoor shooting season	13.8*		6.9–28.8		22	
		Airgun Shooters before indoor shooting season	9.1*		4.7–17.9		21	
		Airgun Shooters after indoor shooting season	8.4*		2.0–22.2		21	
		Archers before shooting season	6.1*		2.7–9.2		13	
		Archers after shooting season	5.6*		3.1–8.7		13	
		Shooter: shot 600 rounds 3 times per week for past 4 months	43.5			United Kingdom	1	White (1996) [97]
	Indoor/Outdoor							
		Shooter	45	6.3	35–47	Connecticut, USA	6	Cook et al. (2015) [98]
		Irregular Frequency Indoor and Outdoor Shooter	6.7			Australia	1	Gulson et al. (2002) [99]
		Archers who also shot guns	7.8	10.26		South Africa	15	Mathee et al. (2017) [15]
		Gun shooters only (excluding archers and employees)	12.13	10.18			62	
		Gun shooters who also work at the shooting range	16.3	7.83			8	
		All gun shooters (including archers and employees)	11.9	10.15			85	
		Less than monthly	8.4	5.5			23	
		> monthly, but less than weekly	11.8	8.7			35	
		> weekly, but < 3 times per week	14.3	15.2			21	
		>3 times per week	12.5	9.7			7	
		Pistol Sport Shooters	10.76	8.28		Helsinki, Finland	20	Asa-Mäkitaipale et al. (2009) [42]
	Outdoor							
		Archery Range	4.5	2.7		South Africa	14	Mathee et al. (2017) [15]
		Shooting Range #1	7	4.3			12	
		Practised archery only	3.3	2.25			31	

## Results - blood lead levels at firing ranges

The search identified 36 articles originating from 15 countries around the world published between 1975 and 2016 that included BLLs of shooters. Over half of the reports were from the U.S. The articles describe BLLs of law enforcement personnel, high school shooting coaches, and family members ranging from as young as 1-year-old to adult men and women.

### Summary of blood lead levels reported

Data from collected studies reveals the widespread occurrence of BLL by occupational and recreational shooters. The vast majority of these articles reported at least one BLL that exceeded 10 μg/dL. About half of the studies further reported BLLs exceeding 20 μg/dL (18 articles), exceeding 30 μg/dL (17), and even exceeding > 40 μg/dL (15). Indeed, all 36 of the articles indicated BLLs of shooters exceeded 2 μg/dL. Considering that the geometric mean BLL of the U.S. adult population in 2009–2010 was 1.2 μg/dL [34], the BLLs among shooters provide stark evidence of significant exposure, particularly to recreational shooters who do not typically self-screen for BLL. Several key characteristics about BLLs and exposure variables arising from shooting are gleaned from the literature.

#### Baseline and post-shooting blood lead level relationships

Several studies focused on before-after comparisons of shooters, particularly shooters in military and police occupations, and found marked increases in BLL resulting from firing range activities. Tripathi et al. (1989) [9] measured BLLs in police cadets before, and 1, 2 and 5 days after starting shooting practice, and 69 days after the start of shooting. At 69 days after the start of shooting, the BLLs of the cadets remained above baseline levels prior to shooting. Rocha et al. (2014) [35] conducted a study of BLLs of police cadets before a shooting course and 3 days after the cessation of the shooting course. The mean BLL of cadets increased from 3.3 μg/dL (95% CI = 3.0–3.6 μg/dL) before the course to 18.4 μg/dL (95% CI 16–21 μg/dL) 3 days after completion of the course. In all cases the BLL increased significantly after the course (*p* <0.001). Within 3 days, the BLLs of the course instructors increased from 3.6 μg/dL to 22.1 μg/dL in one case and from 7.7 μg/dL to 18.3 μg/dL in another. Fischbein et al. (1979) [36] conducted a study of 23 firearms instructors and reported that the BLLs increased measurably after firearms training. Vivante et al. (2008) [37] reported a statistically significant (*p* <0.001) increase in BLLs of 29 Israeli soldiers from a baseline of 10.3 ± 2.0 μg/dL to 18.9 ± 3.6 μg/dL six weeks after training.

#### Decline in blood lead levels after shooting events

Several studies provide insight into the decline in BLLs following shooting events. Goldberg et al. (1991) [38] observed that average BLLs in 7 firing range instructors decreased from 45 μg/dL to 31 μg/dL 6 months after a training event. George et al. (1993) [39] observed that average BLLs in 52 small bore rifle recreational shooters declined from 54.7 μg/dL at the end of the indoor season to 33.1 μg/dL in 37 of the shooters by the preseason of the following year. Smart et al. (1994) [40] observed that average BLLs of 20 howitzer operators declined from 20.1 μg/dL to 11.9 μg/dL in 12 operators 8 weeks after ending the firing exercise. Tripathi et al. (1989) [9] observed that BLLs of 7 outdoor firing range police cadets had a baseline average of 6 μg/dL prior to commencing shooting training and an average BLL of 15 μg/dL at the end of training 5 days later. At follow up 69 days after training, the average BLL was 9 μg/dL. Thus, the results indicate that BLLs following shooting events can remain elevated for a considerable time after cessation of shooting, especially for participants with higher BLLs.

### Association between blood lead levels and bullets fired, caliber of weapon, copper jacketed or unjacketed bullets and air lead levels

Several characteristics such as shooting frequency, caliber of the gun, type of bullet, and air lead at firing ranges have been studied. Each of these variables relate to BLLs and can also be associated with environmental issues at firing ranges.

#### BLLs and frequency of shooting activity

Most studies reviewed indicate a strong positive correlation between the use frequency of shooters at firing ranges and their BLLs. Madrid et al. (2016) [41] reported that BLLs were higher (*p* <0.001) in individuals who participated in greater than 12 shooting practice sessions per year (8.3 ± 2.4 μg/dL) compared with controls who shot less than 12 times per year (5.2 ± 2.5 μg/dL). Tripathi et al. (1989) [9] observed a positive association between the total number of rounds fired and BLLs (r = 0.84; *p* <0.02) and personal-breathing-zone air lead levels (*r* = 0.92; *p* <0.001). Air lead levels were also correlated with BLLs (*r* = 0.85; *p* <0.02). Asa-Mäkitaipale et al. (2009) [42] reported a correlation between BLLs and bullets fired during the last month (*r* = 0.71; p 0.001) and the past year (*r* = 0.55; p 0.012). Betancourt (2012) [43] observed a linear relationship between air lead exposure and total number of rounds fired by caliber of weapon used.

#### Blood lead and gun caliber

Relationships between BLL and caliber of firearms have also been described. Demmeler et al. (2009) [44] observed that the larger the caliber of the weapon, the higher the shooters BLL. The following median BLLs were reported: airguns – 3.3 μg/dL (range 1.8–12.7 μg/dL); 0.22 caliber weapons – 8.7 μg/dL (range 1.4–17.2 μg/dL); 0.22 caliber and large caliber handguns (9 mm or larger) – 10.7 μg/dL (range 2.7–37.5 μg/dL); and large caliber handguns – 10.0 μg/dL (range 2.8–32.6 μg/dL). Demmeler et al. [44] also reported that shooters belonging to the International Practical Shooting Confederation (IPSC) had the highest median BLL of 19.2 μg/dL. Additionally, studies indicated a positive correlation between cumulative air lead exposure in firing ranges and BLL of shooters [40, 45].

#### BLLs and copper jacketed vs. unjacketed bullets

Tripathi et al. (1991) [46] compared the BLLs in firearm instructors using copper jacketed and non-jacketed bullets. One shooting instructor exhibited BLLs of 24.0 μg/dL and 22.0 μg/dL using non-jacketed bullets and copper-jacketed bullets, respectively. A second instructor exhibited BLLs of 14.1 μg/dL and 13.0 μg/dL using non-jacketed bullets and copper-jacketed bullets, respectively.

#### BLLs and air lead

Elevated BLLs especially arising from indoor firing ranges are the result of the greater absorption of lead from inhalation compared with ingestion and dermal absorption. For example, the amount of absorption of ingested lead by adults under non-fasting conditions ranges from 3 to 10% and in young children from 40 to 50% whereas inhaled lead lodging deep in the respiratory tract seems to be absorbed equally and totally, regardless of chemical form [47]. As shooting involves generation of extremely fine particles and gases, the high rate of absorption logically results in elevated BLLs. Outdoor ranges, presumably well-ventilated by natural flow and large air volumes, do not necessarily prevent lead exposure from shooting activities. The following sections discuss the implications of the results.

## Discussion

The results in Table 1 must be evaluated in the context of BLL recommendations, special need populations, air lead measured at firing ranges, and prevention. The use of ventilation to manage exposure at firing ranges and prevent lead exposure of shooters is appraised.

### Blood lead level recommendations from public and occupational health communities

Several United States (US) governmental agencies have developed recommendations regarding BLLs. The Centers for Disease Control and Prevention (CDC) makes health recommendations to protect public health whereas the National Institute for Occupational Safety and Health (NIOSH) and the Occupational Safety and Health Administration (OSHA) focus on worker health. The trend for BLL recommendations has been declining over several decades since regulations were first established.

#### CDC and NIOSH

The CDC [34] makes the following statement regarding recommended BLLs in adults:

“*In 2015, NIOSH designated 5 μg/dL (five micrograms per deciliter) of whole blood, in a venous blood sample, as the reference blood lead level for adults. An elevated BLL is defined as a BLL ≥5 μg/dL. This case definition is used by the ABLES program, the Council of State and Territorial Epidemiologists (CSTE), and CDC’s National Notifiable Diseases Surveillance System (NNDSS). Previously (*i.e. *from 2009 until November 2015), the case definition for an elevated BLL was a BLL ≥10 μg/dL. The U.S. Department of Health and Human Services recommends that BLLs among all adults be reduced to <10 μg/dL. The U.S. Occupational Safety and Health Administration (OSHA) Lead Standards require workers to be removed from lead exposure when BLLs are equal or greater than 50 μg/dL (construction industry) or 60 μg/dL (general industry) and allow workers to return to work when the BLL is below 40 μg/dL….*

*OSHA Lead Standards give the examining physician broad flexibility to tailor special protective procedures to the needs of individual employees. Therefore, the most current guidelines for management of lead-exposed adults should be implemented by the medical community at the current CDC/NIOSH reference BLL of 5 μg/dL. Recommendations for medical management are available from the Association of Occupational and Environmental Clinics, California Department of Public Health, and the Council of State and Territorial Epidemiologist (CSTE) Occupational Health Surveillance Subcommittee*.”

#### Council of state and territorial epidemiologists (CSTE)

The CSTE is an organization of member states and territories representing public health epidemiologists in the United States. The CSTE [48] makes the following recommendations actions for various blood lead levels in adults (Table 2):

**Table 2:** Council of State and Territorial Epidemiologists Management Recommendations for Adult Blood Lead Levels

Blood Lead Level (μg/dL)	Management Recommendations
<5	No action needed
	Monitor BLL if ongoing exposure
5–9	Discuss health risks
	Minimize exposure
	Consider removal for pregnancy and certain medical conditions
	Monitor BLL
10–19	Decrease exposure
	Remove from exposure for pregnancy
	Consider removal for certain medical conditions or BLL >
	10 for an extended period of time
	Monitor BLL
20–29	Remove from exposure for pregnancy
	Remove from exposure if repeat BLL in 4 weeks remains > 20
	Annual lead medical exam recommended
30–49	Remove from exposure
	Prompt medical evaluation
50–79	Remove from exposure
	Prompt medical evaluation
	Consider chelation with significant symptoms
≥80	Remove from exposure
	Urgent medical evaluation
	Chelation may be indicated

Ideally, recommendation triggering immediate cessation of exposure at shooting ranges should not be based on a single blood lead level measurement. The duration of an elevated BLL over multiple BLL measurements should determine the nature of the intervention. Current public health recommendations call first for education and attention to risk factors that can mitigate future exposures.

#### Occupational health and safety administration (OSHA) and the new science-based recommendations

For occupational shooters and firing range workers, the U.S. OSHA Lead Standards require general industry workers to be removed from lead exposure when BLLs are equal or greater than 60 μg/dL, and allows them to return to work when their BLL is below 40 μg/dL [34]. Based on the recommended BLLs by the CDC/NIOSH [34], the CSTE [48] and the comprehensive compilation of health effects of low level lead exposure by the NTP [30], the OSHA regulation that allows workers to return to work with BLLs greater than 40 μg/dL seems nonsensical as a health risk avoidance guideline, and should be lowered in line with the Council of State and Territorial Epidemiologists (CSTE) recommendations, as shown in Council of state and territorial epidemiologists (CSTE).

### Special needs of women and children

Lead exposure of women and children have special characteristics that must be taken into account. The needs relate to the effect of lead on future generations. For women the needs are related to the effect of lead on the developing fetus and post-natal exposure associated with breast-feeding. For children the special needs for low exposure are related to the extraordinary sensitivity of the developing organs of children. These concerns indicate the need for a margin of safety.

#### The special lead risks of women

The risk to women exposed to lead at firing ranges is of particular concern because, once absorbed, a proportion of the lead is deposited in the skeleton and more than 90% of lead in adults is stored in their bones. Bone storage takes place because due to their similar ionic radius and charge lead is substituted for calcium. Furthermore, when a woman becomes pregnant the fetus requires calcium and, depending on the dietary intakes, a proportion of calcium is derived from remodelling of the bones. Skeletal lead stores are released from the remodelling exposing the fetus during critical development windows [49–51]. Even modestly elevated BLL’s have been associated with serious neurological disorders such as autism [52]. Lead released from a woman’s bones during pregnancy is associated with foetal developmental problems [53]. Another consideration for female shooters is that when their BLL becomes elevated, they can pass the lead on to their children through breast milk [54, 55]. Given the known lead contamination at firing ranges, intending-to- conceive, pregnant women, and nursing mothers should curtail exposure from shooting activities (employed in the security, military and police, and recreational shooters) and observe precautionary prevention.

#### Health risks related to children shooters

The CDC (2005) [56] reported that children (aged 7-18) shooting bullets at multiple firing ranges in Alaska exhibited highly elevated BLLs (see Table 1). Shannon (1999) [57] reported that children (aged 14–16) who were competitive marksmen exhibited an average BLL of 21.3 μg/dL (range 18–28 μg/dL). Blood lead levels observed in children from shooting activities are within the range known to cause long-term detrimental health effects [30]. Exposure of young females to lead is of particular concern because it is stored in their bones and can then be transferred to their developing fetus many years later when they become pregnant [49–51].

#### Health-related lead issues and law enforcement personnel

Law enforcement includes a number of services to protect and ensure the safety of citizens and the community. The public requires law enforcement personnel to be “calm, cool and collected” when in service conducting their duties. However, the adverse health effects, especially on the nervous system that are associated with elevated BLLs arising from firearm use are inconsistent with these ideals.

### Air lead levels at firing ranges

The OSHA 8-h air lead time weighted average (TWA) action level is 30 μg/m^3^ and the OSHA permissible exposure limit (PEL) is 50 μg/m^3^ [58]. The California Department of Public Health Occupational Lead Poisoning Prevention Program (CDPH-OLPPP) recommended 8 h TWA PEL is 0.5 to 2.1 μg/m^3^ [59]. Based on this guideline, the CDPH-OLPPP states “*At a PEL of 0.5 μg/m* ^*3*^ *, 95% of workers would have a BLL less than 5 μg/dL over a 40 year working lifetime. At a PEL of 2.1 μg/m* ^*3*^ *, 95% of workers would have a BLL less than 10 μg/dL and 57% would have a BLL less than 5 μg/dL over their working lifetime*.” Wang et al. (2016) [60] conducted a review of studies of airborne lead concentrations and possible exposure at firing ranges (Additional file 1). Wang et al. [60] found that the OSHA 8 h TWA PEL is exceeded in many studies, and even more noteworthy, the California PEL is exceeded in *all* of the studies. It must be noted that the recommended PEL and action levels are not the only paths to controlling lead exposures.

### Biomonitoring and primary prevention

Kosnett et al. (2007) [61] recommend that: “*individuals be removed from occupational lead exposure if a single blood lead concentration exceeds 30 microg/dL or if two successive blood lead concentrations measured over a 4-week interval are > or = 20 microg/dL. Removal of individuals from lead exposure should be considered to avoid long-term risk to health if exposure control measures over an extended period do not decrease blood lead concentrations to < 10 microg/dL or if selected medical conditions exist that would increase the risk of continued exposure*.” A more conservative approach are the recommendations by CSTE in Council of state and territorial epidemiologists (CSTE). A critical issue is that biomonitoring is not primary prevention. Biomonitoring only assesses the degree of exposure and potential health damage after exposure has taken place. Primary prevention requires curtailing lead exposure and maintenance of air quality. Several steps have been proposed above to minimize lead exposure. Recommendations to prevent occupational lead poisoning by shooters are provided by U.S. Government [62]. The recommendations appear as topics in school rifle team programs [63].

One of the challenges in a biomonitoring program is the frequency which shooters should have their BLLs monitored. The Australian organisation Safe Work Australia has recently carefully made recommendations for multiple scenarios of blood lead testing frequency for workers exposed to lead in the work place [64]. Similar BLL testing frequency recommendations could be adopted for shooters exposed to lead in occupational settings such as law enforcement, military, security and shooting range workers. Recreational shooters that shoot frequently could voluntarily use these blood lead testing frequency recommendations as a guide if they wanted to protect their health.

#### Potential health risks from ‘take home lead’

In contrast to occupational environments where work clothes should not be taken home, lead dust can adhere to shooters clothes and potentially contaminate vehicles and homes. The CDC (1996) [65] measured carpet dust lead concentrations in FBI student dormitory rooms and in 14 non–student dormitory rooms at a firing range and training facility. They observed that student dormitory rooms had significantly higher lead levels than non–student dormitory rooms, suggesting that the FBI students were contaminating their living quarters with lead. ‘Take home lead’ has been described mostly for occupational settings [66–68] but given the fine particle nature and lead concentrations of dust associated with shooting, the ‘take home lead’ pathway of exposure from shooting must be recognized and curtailed.

#### Prevention of lead aerosols with ventilation improvements

The air lead table from Wang et al. 2016 [60] (Additional file 1) and the National Research Council [69] are the only compilations of air Pb levels at shooting ranges that were identified. Wang et al. do not discuss the ventilation practices in the various studies that may account for lower air lead levels. A 1975 NIOSH study found that at all 9 ranges studied, the air lead guideline was exceeded at the time (200 μg/m^3^) [70]. A 2009 NIOSH review describes a case study on air lead exposure of law enforcement trainees and reports that the mean airborne lead concentration of >2000 μg/m^3^ was reduced by 94–97% to 60–120 μg/m^3^ but this was still above the OSHA PEL of 50 μg/m^3^. Commercial ventilation companies claim they can meet guidelines (i.e. Camfil air filters) but no published studies supporting this achievement at firing ranges were located.

There is a “lack of evidence” gap in the literature demonstrating that ventilation systems can maintain air lead levels at indoor ranges below the current OSHA (50 μg/m^3^) or California (0.5–2.2 μg/m^3^) guideline. The literature gap raises questions about whether or not the guidelines can actually be achieved, especially the California guideline. Further, as discussed in Special needs of women and children, meeting the guideline does not necessarily provide a margin of safety from lead exposure.

#### Primary prevention requires eliminating lead in primers and bullets

Lead from projectile primers is a significant proximal source of lead exposure and uptake. The development of primers is described by Brede et al. [71]. During the 19^th^ century primers were composed of mercury fulminate; however, the mercury fulminate was found to be too toxic to shooters. In the early 20^th^ Century, Dynamit Nobel developed the primer SINOXID which was formulated with lead and became a universal primer. By “…the 1960s exposure of shooters and firing range supervisors to lead reached intolerably high levels, as evidenced by the elevated blood lead values [71].” Dynamit Nobel developed SINTOX, a Pb-free (as well as Sb and Ba free) primer [71]. However, the results of some tests of the lead-free primers have proven disappointing, with significant variations in ignition timing, peak blast pressure, higher barrel frictions, and reliability in different climate conditions, compared with their lead-based equivalents [72]. The performance of lead-free primers are being tested by the U.S. Department of Defense (DoD) and North Atlantic Treaty Organisation (NATO) to reduce exposure of personnel to known lead sources [73].

Despite the critical observations, there is lead-free ammunition on the market. SINTOX is NATO approved and outlets for lead-free ammunition are available [74, 75]. Some states are taking the issue seriously and require lead-free (or non-toxic “NT”) ammunition at firing ranges [76]. Widespread acceptance of the need to replace lead must take place, and until this happens one of the most significant health risks to shooters will remain lead-rich primers.

“Green bullets” have also been proposed as a preventative measure that could minimize lead exposure to participants and the environment. These bullets consist of copper rather than lead bullets. Bismuth has been proposed as a substitute for lead bullets but its environmental health impacts are poorly understood [77]. It is clear that firing lead-free bullets results in dramatic decreases in airborne lead exposures at firing ranges [78]. The use of copper-jacketed lead bullets does not appear to be a solution to a reduction in lead exposure because it results in only minor reductions in BLLs (see Tripathi et al. (1991, Table 1) [46]. The United States Department of Defence (DoD) is aware of the health threat posed by lead exposure from small arms [69] and efforts are underway to test and replace lead in both primer and bullets [73, 79].

Table 1 provides evidence-based information about the BLL sensitivity of shooters to lead dust at firing ranges. The major gap in preventing risk of lead exposure at firing ranges are the fundamental lead-bearing materials used for the explosive power and bullet projectiles. Primary prevention requires eliminating all lead materials in primers and bullets in order to end the dispersal of lead dust at firing ranges.

## Conclusions

Shooting lead bullets at firing ranges results in elevated BLLs at concentrations that are associated with a variety of adverse health outcomes and the topic of health risk is an ongoing topic of study. Of major concern is the number of women and children among recreational shooters, who are not afforded similar health protections as occupational users of firing ranges. Nearly all BLL measurements compiled in the reviewed studies exceed the level of 5 μg/dL recommended by the U.S. CDC/NIOSH, and thus firing ranges, regardless of type and user classification, constitute a significant and currently largely unmanaged public health concern. Primary prevention of this risk requires development of lead-free primers and projectile bullets. Prevention includes better oversight of ventilation systems in indoor ranges and development of airflow systems at outdoor ranges, protective clothing that is changed after shooting, and cessation of smoking and eating at firing ranges. The mismatch between what is recommended for individuals by the U.S. CDC is in stark contrast to the allowable levels for occupational exposure, and there are no real systematic biomonitoring programs for firing range users to measure cumulative health effects caused by persistent low and even high-level lead exposure. Recreational shooters and the general public are provided no legal protections from lead exposures at firing ranges. In conclusion, while the past two decades have brought substantial improvements in analytical capabilities to detect lead in humans the literature evidence indicates that we fall far short of human health safety criteria in firing ranges of all types, and among occupational and recreational shooters.

## Additional file

Additional file 1: Summary of Studies on Airborne Lead Exposure and Concentration from Shooting Activities, by Chronological Orders (modified from Wang et al., 2016). (DOCX 19 kb)

## Abbreviations

ABLES: Adult blood lead and epidemiology surveillance
BLLs: Blood lead levels
CDC: Centers for disease control and prevention
CDPH-OLPPP: California department of public health occupational lead poisoning prevention program
CSTE: Council of state and territorial epidemiologists
DOD: Department of defence
IPSC: International practical shooting confederation
IQ: Intelligence quotient
NATO: North atlantic treaty organisation
NIOSH: National institute for occupational safety and health
NNDSS: National notifiable diseases surveillance system
NSSF: National sports shooting foundation
NT: Non-toxic
NTP: National toxicology program
OSHA: Occupational safety and health administration
Pb: Lead
PEL: Permissible exposure level
US: United States
USGS: United States geological survey
