# Supplementary material for: Lead exposure at firing ranges—a review
Source: Environ Health. 2017 Apr 4;16:34. doi: 10.1186/s12940-017-0246-0 (PMC5379568; doi:10.1186/s12940-017-0246-0)
Supplement: Additional file 1: — Summary of Studies on Airborne Lead Exposure and Concentration from Shooting Activities, by Chronological Orders (modified from Wang et al., 2016). (DOCX 19 kb) [file 12940_2017_246_MOESM1_ESM.docx]

| **Supplement 1- Summary of Studies on Airborne Lead Exposure and Concentration from Shooting Activities, by Chronological Orders (modified from Wang et al., 2016).** | | | | |
| --- | --- | --- | --- | --- |
| **Study** | **Studied Personnel** | **Indoor/Outdoor/**  **Other Firing ranges** | **Firearm/Ammunition** | **Airborne Lead Levels**^a^ |
| Fischbein et al. (1979) | Law enforcement officers | Indoor | n.s. b | 45–900 µg/m^3^ (with a peak exposure of 3,750 mg/m3 during firing) |
| Muskett and Caswell (1980) | Range workers | Indoor | Rifle | 55–113 µg/m^3^ |
| Novotny et al. (1987) | Ranger workers | Indoor | n.s. | 2.7–90.5 µg/m^3^ |
| Valway et al. (1989) | Law enforcement officers | Indoor and outdoor | n.s. | 2,000 µg/m^3^ (short-term) |
|  |  |  |  | 304 µg/m^3^ (Corrected 8-h TWA by Stern) |
| Goldberg et al. (1991) | Law enforcement officers | Indoor and outdoor | Lead and lead-free | 460–510 µg/m^3^ (lead, 3-h TWA) |
|  |  |  |  | 100–170 µg/m^3^ (lead-free, 3-h TWA) |
| Bonanno et al. (2002) |  | Outdoor | Lead | 286 µg/m^3^ (22 calibre - summer) |
|  |  |  |  | 579 µg/m^3^ (center fire - summer) |
|  |  |  |  | 235 µg/m^3^ (22 calibre - winter) |
|  |  |  |  | 1158 µg/m^3^ (center fire - winter) |
| Mancuso et al. (2008) | Special Operations Soldiers | Indoor | Lead | 980 - 1900 µg/m^3^ TWA (unspecified time) |
|  |  | Indoor | Lead | 60-220 µg/m^3^ TWA (unspecified time) |
|  |  | Outdoor | Lead | 100-230 µg/m^3^ TWA (unspecified time) |
| Chen and Brueck (2011) | Instructors | Outdoor | n.s. | n.d.-15 µg/m^3^ |
| Ramsey and Niemeier (2011) | Range workers and Indoor shooters | Indoor | n.s. | n.d.-96 µg/m^3^ (instructor) |
|  |  |  |  | 42–340 µg/m^3^ (shooters, task-based) |
|  |  |  |  | 3,200 µg/m^3^ (technician, task-based) |
| Scott et al. (2005) | Indoor Law Enforcement | Indoor | Lead | 20.6 μg/m^3^ to 99.0 μg/m^3^ (8-h TWA), |
|  |  |  |  | 26 μg/m^3^ to 287 μg/m^3^ (8-h TWA) |
| Betancourt (2012) | Special Operations Soldiers | Outdoor | Lead | 48 μg/m^3^ (8-h TWA) |
| Scott et al. (2012) | Law enforcement officers | Indoor | n.s. | 60–3,200 µg/m^3^ (short-term) |
|  |  |  |  | 20.6–2,897 µg/m^3^ (8-h TWA) |
|  |  |  |  | 60–2,000 µg/m^3^ (area short-term) |
|  |  |  |  | 43.8–350 µg/m^3^ (area 8-h TWA) |
| Ramsey et al. (2013) | Range workers and Indoor shooters | Indoor | n.s. | 0.15–3.8 µg/m^3^ (instructor) |
|  |  |  |  | 1.5–9.0 µg/m^3^ (shooters, task-based) |
|  |  |  |  | n.d.–330 µg/m^3^ (technician, task-based) |
| Brueck et al. (2014) | Military personnel | Indoor | n.s. | n.d.–26 µg/m^3^ (short-term) |
| CDC (2014) | Range workers | Indoor | n.s. | 5.5–19 µg/m^3^ (showroom worker) |
|  |  |  |  | 54–64 µg/m^3^ (range cleaning, short-term) |
| Wingfors et al. (2014) | n.s. | Chamber | Lead-free | 7.3–7.4 µg/m^3^ per round fired (emission factors) |
| Lach et al. (2015) | n.s. | Indoor/outdoor | Lead and lead-free | 2.2–70 µg/m^3^ (area) |
| ^a^ Results are presented in 8-h TWA personal exposure unless noted otherwise (short-term/task-based, full-shift, area, emission factors). | | | | |
| ^b^ n.s.: not specified, n.d.: not detected. | | | | |
